# Characteristics and mechanisms of nickel adsorption on biochars produced from wheat straw pellets and rice husk

**DOI:** 10.1007/s11356-017-8847-2

**Published:** 2017-03-31

**Authors:** Zhengtao Shen, Yunhui Zhang, Oliver McMillan, Fei Jin, Abir Al-Tabbaa

**Affiliations:** Geotechnical and Environmental Research Group, Department of Engineering, University of Cambridge, Cambridge, CB2 1PZ UK

**Keywords:** Biochar, Standardised production, Adsorption studies, Adsorption capacity, Adsorption mechanisms, Nickel

## Abstract

The adsorption characteristics and mechanisms of Ni^2+^ on four-standard biochars produced from wheat straw pellets (WSP550, WSP700) and rice husk (RH550, RH700) at 550 and 700 °C, respectively, were investigated. The kinetic results show that the adsorption of Ni^2+^ on the biochars reached an equilibrium within 5 min. The increase of the solid to liquid ratio resulted in an increase of Ni^2+^ removal percentage but a decrease of the adsorbed amount of Ni^2+^ per weight unit of biochar. The Ni^2+^ removal percentage increased with the increasing of initial solution pH values at the range of 2–4, was relatively constant at the pH range of 4–8, and significantly increased to ≥98% at pH 9 and stayed constantly at the pH range of 9–10. The calculated maximum adsorption capacities of Ni^2+^ for the biochars follow the order of WSP700 > WSP550 > RH700 > RH550. Both cation exchange capacity and pH of biochar can be a good indicator of the maximum adsorption capacity for Ni^2+^ showing a positively linear and exponential relationship, respectively. This study also suggests that a carefully controlled standardised production procedure can make it reliable to compare the adsorption capacities between different biochars and investigate the mechanisms involved.

**Electronic supplementary material:**

The online version of this article (doi:10.1007/s11356-017-8847-2) contains supplementary material, which is available to authorized users.

## Introduction

Biochar is produced by heating biomass (wood, grass, crop residues, manure and sewage sludge) under oxygen-limited conditions in a process called pyrolysis (Lehmann 2007; Sohi 2012). The incomplete carbonisation of the biomass during the pyrolysis process changes the properties of the feedstock, resulting in biochar with a porous structure, high surface area, high pH, active functional groups and a graphite-like aromatic structure (Keiluweit et al. 2010; Manyà 2012; Ronsse et al. 2013; Xin et al. 2015). These properties result biochar with high adsorption capacities for heavy metals (Keiluweit et al. 2010; Beesley et al. 2011; Inyang et al. 2015). Therefore, the application of biochar to contaminated soils has gained increasing attention from scientists and engineers due to the predicted ability of biochar to immobilise heavy metal contaminants, in addition to its other benefits including low cost, carbon storage, greening and sustainability (Zhang et al. 2013; Sizmur et al. 2015; Shen et al. 2016).

Prior to field application in soil remediation, it is important to understand the adsorption characteristics and mechanisms of biochar for heavy metals in order to aid engineering design. Biochars with high adsorption capacities for heavy metals are preferred however site-specific factors such as heavy rainfall and changing soil pH should also be considered. A number of laboratory studies have been carried out to investigate the characteristics of heavy metal adsorption on biochar (Inyang et al. 2015); however, the biochar production conditions among different studies vary significantly, resulting in significant variations in the adsorption properties among biochars. It is not always possible to make relevant comparisons to adsorption capacities in the literature even for biochars derived from the same feedstock, as the production process may be differently controlled. It is therefore important to standardise the production process of biochar for laboratory adsorption studies so that the adsorption capacities of biochars can be reasonably compared across different studies; the adsorption mechanisms can be critically analysed; and the biochars which are most suitable for a specific contaminated site can be identified.

In addition, the characteristics of heavy metal adsorption on biochars produced from different feedstocks at different temperatures still need to be investigated as an addition to the existing biochar literature, to explore the adsorption mechanisms and aid the potential application of biochar in soil remediation. For instance, although wheat straw derived biochars exhibited a strong ability to remove methylene blue (Liu et al. 2012a) and to enhance crop yields (Qu et al. 2012), no existing studies have investigated the adsorption characteristics or mechanisms of Ni^2+^, Cu^2+^ or Pb^2+^ on wheat straw-derived biochars to the best of the author’s knowledge. Likewise, the adsorption characteristics of Ni^2+^ on biochar have not been studied.

In this study, four biochars, recommended by the UK Biochar Research Centre (UK Biochar Research Centre, 2016) as standard biochars, were produced from two plant-based feedstocks at two different temperatures at a standardised procedure. This study aims to (1) investigate the adsorption characteristics of heavy metals on these standard biochars, (2) identify the biochars which are suitable for future application to a contaminated site, and (3) understand the link between the physicochemical properties of these biochars and their adsorption capacities. Ni^2+^ was selected as the target metal ion in the adsorption studies as it exists in high concentrations at an existing contaminated site of interest to the authors (Shen et al. 2016). The carefully controlled production procedure of the biochars with high reproducibility (UK Biochar Research Centre, 2016) provides the reliability to compare the adsorption characteristics among the biochars and investigate the adsorption mechanisms involved.

## Materials and methods

### Biochar

The standard biochars were derived from wheat straw pellets (WSP) and rice husk (RH). Wheat and rice are two of the main crops across the world (Lal 2005), and a large number of their agricultural residues are generated every year, e.g. tonnes of unused wheat straw residues in North America (Alemdar and Sain 2008) and millions of tonnes of rice husks in China (Armesto et al. 2002). Therefore, the production of biochars from wheat straw and rice husk can both help deal with the agricultural wastes and aid the large-scale applications in soil remediation due to the high availability. WSP and RH were produced at 550 and 700 °C, resulting in four biochars named WSP550, WSP700, RH550 and RH700. The pyrolysis process was carried out by the UKBRC at the University of Edinburgh, and production parameters were carefully controlled (UKBRC 2016), resulting in high reproducibility of the standard biochars. Upon receipt, the biochars were oven dried at 60 °C for 48 h and sieved to a particle size of less than 0.15 mm for further analysis. The cation exchange capacity (CEC) of biochar was determined by a compulsive exchange method based on (Gillman and Sumpter 1986). The surface morphology of the biochar was examined by a scanning electron microscopy (SEM) at 15 kV after coated with gold. The infrared spectrum of biochar was obtained using a Perkin-Elmer Spectrum 100 Fourier transform infrared spectroscopy (FT-IR) spectrometer by taking 16 scans from 4000 to 450 cm^−1^ with a resolution of 1 cm^−1^. Other physicochemical properties of biochar were tested by the UKBRC and can be found from (UK Biochar Research Centre, 2016).

The physicochemical properties of the biochars are shown in Table 1. The physicochemical properties of the biochars are significantly affected by the feedstock and production temperature. For instance, the biochars consist primarily of carbon and ash, but WSP-derived biochars contains significantly higher carbon contents (68.26–69.04%) compared with RH (47.32–48.69%) and lower ash contents (21.25–23.82% and 47.93% for WSP- and RH-derived biochars, respectively). Increasing the production temperature from 550 to 700 °C resulted in a slight decrease of surface area from 26.40 to 23.20 m^2^/g for WSP-derived biochars while a significant increase from 21.10 to 42.00 m^2^/g for RH-derived biochars. WSP-derived biochars showed significantly higher H, O, N and P contents, VM content, H:C and O:C values, pH and CEC, compared with RH at the same production temperature. For the same feedstock, increasing production temperature decomposes the cellulose, hemicellulose and lignin in the raw materials (Keiluweit et al. 2010), leading to lower H, O and N contents, VM content and H:C and O:C values, but higher pH and CEC which are probably due to the accompanied generation of alkaline minerals (Dodson 2011).

**Table 1:** Physicochemical properties of the biochars

	WSP550	WSP700	RH550	RH700
C (%)	68.26	69.04	48.69	47.32
H (%)	2.10	1.18	1.24	0.63
O (%)	6.92	5.30	2.47	2.06
N (%)	1.39	1.32	1.04	0.85
P (%)	0.14	0.25	0.10	0.16
VM (%)	10.55	7.38	7.48	4.99
H:C	0.37	0.20	0.28	0.16
O:C	0.08	0.06	0.04	0.03
Total ash (%)	21.25	23.82	47.93	47.93
pH	9.94	10.03	9.71	9.81
Surface area (m^2^/g)	26.40	23.20	20.10	42.00
CEC (cmol/kg)	7.15	12.50	4.22	5.36
K (%)	1.56	1.47	0.39	0.62
Ni (mg/kg)	1.00	2.50	3.00	2.71

The standard deviations (SD) for CEC were within 0.10–0.23; the SD for other properties can be found from (UKBRC 2016)

*VM* volatile matter, *CEC* cation exchange capacity

### Adsorption studies

Batch adsorption experiments were carried out in polyethylene tubes in a temperature-controlled lab (20 ± 1 °C). Solutions of 0.01, 0.1 and 1 M HNO_3_ and 0.01, 0.1 and 1 M NaOH were used to adjust the initial pH of the solutions where required. For each experiment, the biochar-solution mixture was filtered with a 0.45-μm filter after the designated shaking time. The Ni^2+^ concentration in the collected filtrate was measured by inductively coupled plasma/optical emission spectrometry (ICP-OES) (Perkin-Elmer, 7000DV).

#### Kinetics

In order to assess the adsorption kinetics of the biochars, a certain amount of biochar (0.1 g) was added to 20 mL solutions of 5 mM Ni (NO_3_)_2_ (pH = 5) containing 0.01 M NaNO_3_ (for a stable ionic strength of the solution). The mixture was shaken at 200 rpm for 5, 10, 20 or 30 min or 1, 2, 3, 6, 12 or 24 h. These tests confirmed that the equilibrium adsorption time for all biochars was no longer than 5 min, and longer adsorption time does not affect the equilibrium. Therefore, a reaction time of 24 h was used in the following adsorption studies, which is the same as the previous study (Shen et al. 2015).

#### Influence of solid to liquid ratio

In order to assess the effects of solid to liquid ratio on Ni^2+^ adsorption, a measured amount of biochar (0.1, 0.2, 0.3, 0.4, 0.5, 0.6, 0.7, 0.8, 0.9 or 1 g) was added to 20 mL of 5 mM Ni (NO_3_)_2_ solutions (pH = 5) containing 0.01 M NaNO_3_. The mixture was shaken at 200 rpm for 24 h to reach equilibrium.

#### Influence of pH

In order to assess the effect of initial solution pH on Ni^2+^ adsorption, a certain amount of biochar (0.1 g) was added to 20 mL of 5 mM Ni (NO_3_)_2_ solution (containing 0.01 M NaNO_3_). The initial pH of each solution (before biochar addition) was adjusted to 2, 3, 4, 5, 6, 7, 8, 9 or 10. The mixture was shaken at 200 rpm for 24 h to reach equilibrium. The equilibrium pH of each solution was measured, and the point of zero charge (pH_pzc_) of each biochar was obtained from a plot of initial solution pH against equilibrium solution pH, based on Mohan et al. (2014). In order to separate the precipitated Ni (OH)_2_ due to its solubility at different pHs, the fractions of Ni^2+^ removal caused by this effect were calculated using Visual MINTEQ 3.1 based on the initial concentrations of Ni^2+^, Na^+^ and NO_3_^−^ and the equilibrium solution pH.

#### Equilibrium study

In order to construct an isotherm for each biochar, a certain amount of biochar (0.1 g) was added to 20 mL solutions (pH = 5) containing different Ni^2+^ concentrations (0.1, 0.2, 0.3, 0.5, 1, 2, 3 or 5 mM) and 0.01 M NaNO_3_. The mixture was shaken at 200 rpm for 24 h to reach equilibrium.

#### Calculation

The adsorbed amount of Ni^2+^ on biochar was calculated using Eq. (1).

1 $$ {q}_{\mathrm{e}}=\frac{\left({C}_0-{C}_{\mathrm{e}}\right) V}{W} $$

where *q*_*e*_ is the amount (mmol/g) of Ni^2+^ adsorbed on biochar. *C*_0_ (mM) and *C*_e_ (mM) are the initial and final Ni concentrations in solutions. *V* is the solution volume (L). *W* is the biochar weight (g).

For the influence of solid to liquid ratio and initial solution pH on adsorption, the removal percentage of Ni^2+^ in solution was calculated using Eq. (2). The adsorbed amount of Ni^2+^ per weight unit of biochar was calculated using Eq. (1).

2 $$ {P}_{\mathrm{R}}=\frac{C_{\mathrm{e}}-{C}_0}{C_0} $$

where *P*_R_ is the removal percentage of Ni^2+^. *C*_0_ (mM) and *C*_e_ (mM) are as per Eq. (1).

For the equilibrium study, the experimental data were fitted using Langmuir and Freundlich isotherm models, which are typically used to describe the adsorption isotherms and predict the mechanisms (Foo and Hameed 2010). The Langmuir model is expressed as Eq. (3).

3 $$ {q}_{\mathrm{e}}=\frac{Q_{\max}\mathrm{b}{C}_{\mathrm{e}}}{1+{bC}_{\mathrm{e}}} $$

where *q*_e_ and *C*_e_ are defined as per Eq. (1). Q_max_ (mmol/g) and *b* (L/mmol) are the Langmuir constants representing the maximum adsorption capacity and rate of adsorption.

The Freundlich model is expressed as Eq. (4).

4 $$ {q}_{\mathrm{e}}={K}_{\mathrm{F}}{C_{\mathrm{e}}}^{1/ n} $$

where *q*_e_ and *C*_e_ are defined as per Eq. (1). *K*_F_ (mmol/g) and *n* are Freundlich constants. *K*_F_ is the adsorption capacity of the adsorbent, and *n* indicates the degree of favourable of the adsorption process. 1/*n* ranges between 0 and 1 and is a measure of adsorption intensity or surface heterogeneity. A lower 1/*n* value indicates a greater degree of heterogeneity on the biochar surface.

All experiments were conducted in duplicates. The means and standard deviations were calculated and presented for each experiment. Regression was used to evaluate the fitness of the prediction models to the experimental data in this study using Origin 8.5. The suitability of the model fitting was assessed using *R* ^2^ values and Akaike information criterion (AIC) values.

## Results and discussion

### FT-IR spectra and SEM images

**Fig. 1:**
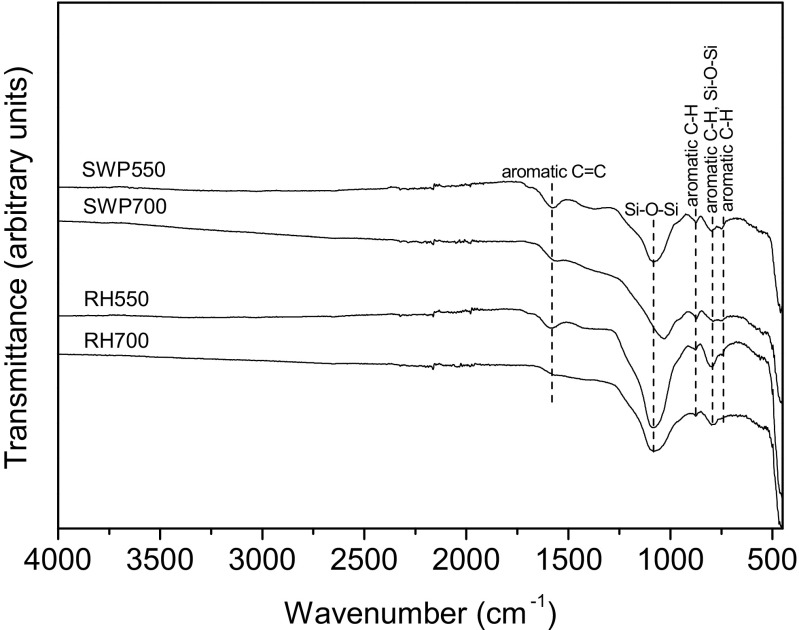
FT-IR spectra of the biochars

**Fig. 2:**
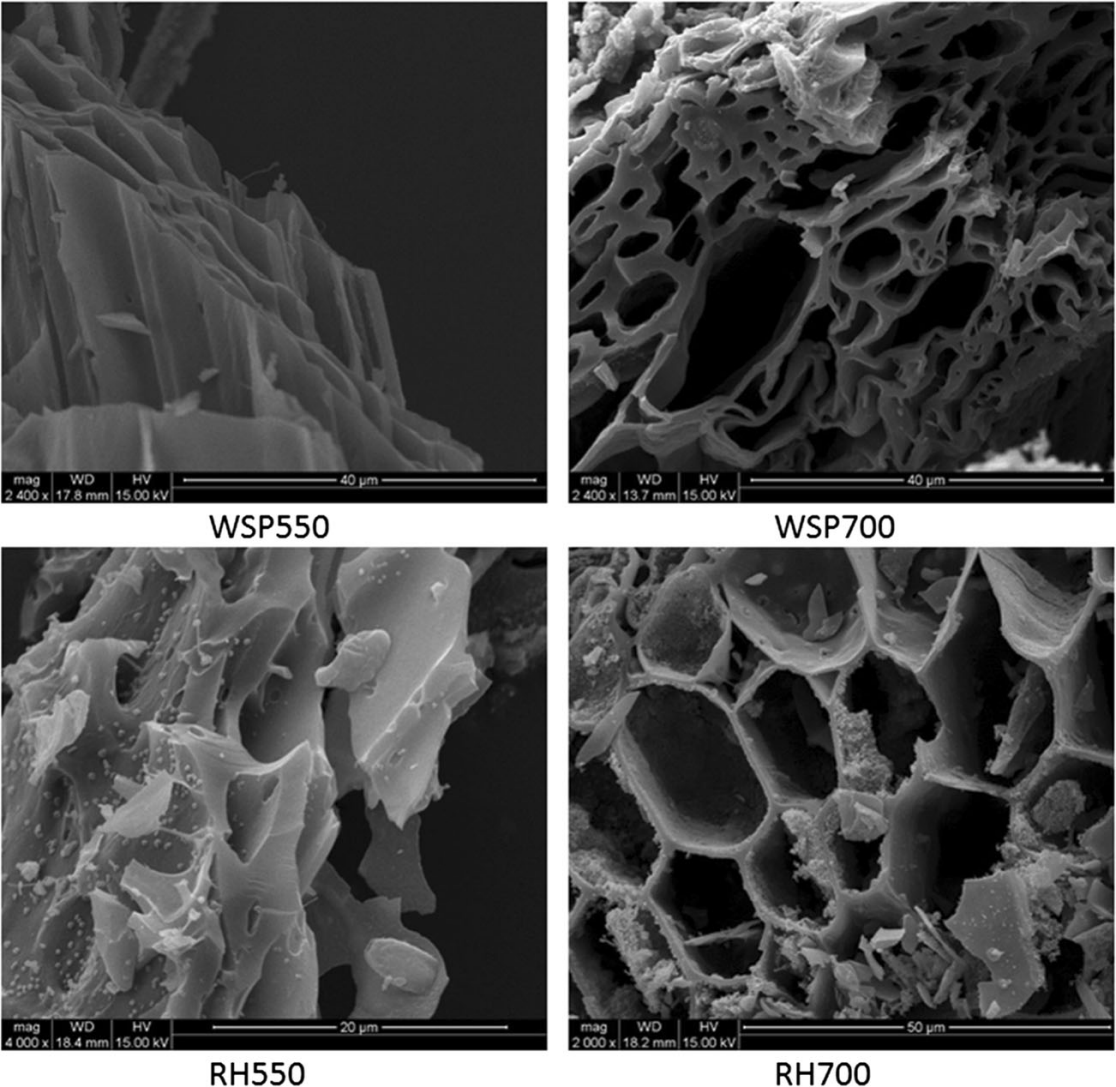
SEM images of the biochars

The FT-IR spectra of the biochars, as shown in Fig. 1, indicate the presence of aromatic C = C stretching (1575–1585 cm^−1^) (Keiluweit et al. 2010), Si-O-Si asymmetric vibration (1035–1080 and 800 cm^−1^) (Liu et al. 2012a; Liu et al. 2012b) and aromatic C-H bending (750, 800 and 875 cm^−1^) (Keiluweit et al. 2010). The stronger Si-O-Si peak of RH-derived biochars compared with that of WSP suggests that RH-derived biochars may have more SiO_2_ contents, which is also reflected by their higher ash contents (Table 1). For the same feedstock, a higher production temperature resulted in a weaker peak for aromatic C = C and C-H. This is due to the condensation of aromatic structure at higher temperatures (Keiluweit et al. 2010). WSP and RH primarily contain cellulose and semi-cellulose (Keiluweit et al. 2010); however, no peaks associated with O-containing functional groups (C = O, C-O, O-H) or aliphatic C-H was observed, indicating a high degree of aromatization of the feedstocks during biochar production (Keiluweit et al. 2010). The SEM images of the biochars (Fig. 2) show the active porous structures on all biochar surfaces. WSP700 exhibited a heterogeneous distribution of pore diameters from <1 to ~20 um. In contrast, RH700 showed relatively homogeneous distribution of macro pores with diameters of ~10–20 um.

### Kinetics

**Fig. 3:**
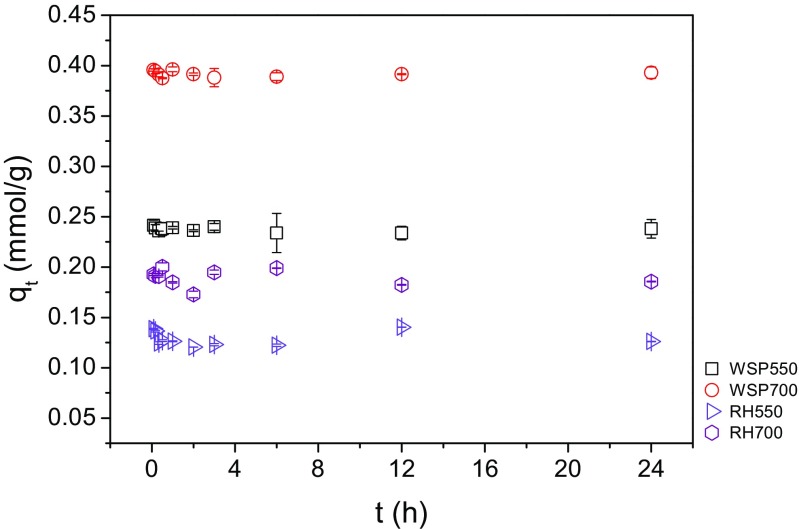
Kinetics of Ni^2+^ adsorption on biochars (*q*_t_—adsorbed amount at time t, t— time) (0.1 g biochar in 20 mL solution (0.01 M NaNO_3_), initial Ni^2+^ concentration 5 mM; reaction temperature 20 °C; initial solution pH 5)

The adsorption profiles of Ni^2+^ to the biochars over time are shown in Fig. 3. The adsorption of Ni^2+^ on all biochars reached equilibriums within 5 min. WSP700 exhibited the highest adsorption capacity (~0.39 mmol/g) at the initial Ni^2+^ concentration of 5 mM, followed by WSP500 (~0.24 mmol/g), RH700 (~0.19 mmol/g) and RH550 (~ 0.13 mmol/g). At the initial Ni^2+^ concentration of 5 mM, WSP-derived biochars exhibited higher adsorption capacities than RH. For the same feedstock, higher production temperature results in a higher adsorption capacity.

The rapid reaction in this study coincides with the findings of Saleh et al. (2016) and Tran et al. (2016). Saleh et al. (2016) used sunflower seed husk biochar (≤0.5 mm) to adsorb Cu^2+^ (Solid to liquid ratio 1:200 g/mL, initial Cu^2+^ concentration 1.5 mM) in aqueous solutions and observed that more than 95% of the totally adsorbed Cu^2+^ at equilibrium was adsorbed on the biochar within 5 min. Similarly, Tran et al. (2016) used orange peel-derived biochar (≤0.71 mm) to adsorb Cd^2+^ (solid to liquid ratio 2 g/L, initial Cd^2+^ concentration 100 mg/L) and found that approximately 80.6–96.9% of the totally adsorbed Cd^2+^ was removed within 1 min. The ability of the biochars to rapidly remove Ni^2+^ from solution and no appearance of desorption within 24 h suggests that there is a potential for the biochars to rapidly treat heavy metals in soil and water. It is of note that the relatively small particle sizes (≤0.15 mm) may also contribute to this rapid reaction, as Rees et al. (2014) found that short-term adsorption of heavy metals (Cu^2+^, Cd^2+^ and Ni^2+^) is highly dependent on intra-particle diffusion, with fine biochar particles (≤0.2 mm) adsorbing heavy metals significantly faster than coarse particles (0.2–2 mm). As the findings (the rapid adsorption of Ni^2+^ on all biochars) from the kinetic study are significant and the initial stage where the *q*_e_ commonly increases with time was not observed, the kinetic data were not fitted using kinetic models for a further analysis.

### Influence of solid to liquid ratio on Ni^2+^ adsorption

**Fig. 4:**
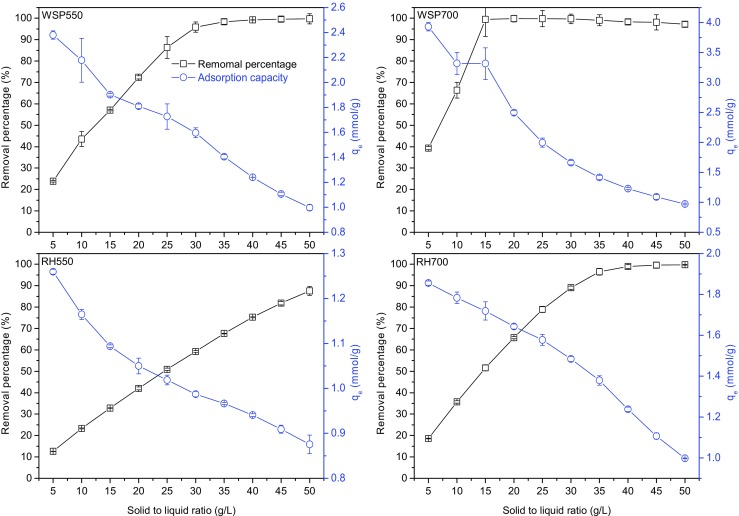
The influence of solid to liquid ratio on Ni^2+^ removal percentage and the adsorbed amount of Ni^2+^ per weight unit of biochar (mmol/g) (initial Ni^2+^ concentration 5 mM in 20 mL solution (containing 0.01 M NaNO_3_), reaction temperature 20 °C, initial solution pH 5, contact time 24 h)

The influences of solid to liquid ratio on Ni^2+^ removal percentage and the adsorbed amount of Ni^2+^ per weight unit of biochar are shown in Fig. 4. The Ni^2+^ removal percentage of WSP700 increased rapidly from 39.30 to 99.45% as the solid to liquid ration increased from 5 to 15 g/L, and stayed constantly when the solid to liquid ratio continued to increase to 50 g/L. The Ni^2+^ removal percentage of WSP550 and RH700 increased from 23.80 and 18.56%, respectively, at 5 g/L to close to ≥99% at 40 g/L and further stayed constantly. The Ni^2+^ removal percentage of RH550 increased with the increasing of solid to liquid ratio, however, did not complete removal (maximum of 87.54%) in the experimental range of 5–50 g/L, due to its low adsorption capacity compared with other biochars, as indicated in Fig. 3. WSP700 completed Ni^2+^ removal (≥99%) using the least solid amount (0.3 g) whereas that for WSP550 and RH700 was much higher (0.8 g), which is also in line with their adsorption capacities observed from Fig. 3. The adsorbed amount of Ni^2+^ per weight unit of biochar decreased as solid to liquid ratio increased from 5 to 50 g/L for all biochars, which is similar to the findings from Meng et al. (2014) and Shen et al. (2015).

### Influence of solution pH on Ni^2+^ adsorption

**Fig. 5:**
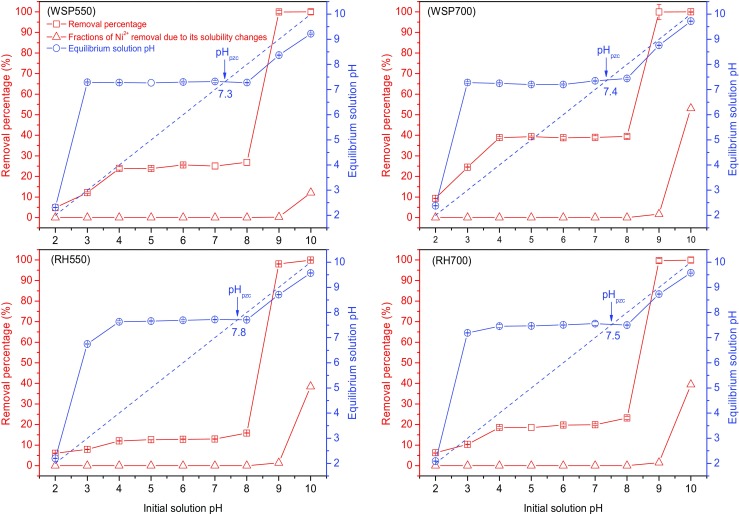
The influence of initial solution pH on the Ni^2+^ removal percentage; the fractions of Ni^2+^ removal caused by the formation of Ni (OH)_2_ due to solubility; and the equilibrium solution pH; the *dashed line* is used to obtain the pH_pzc_ (initial Ni^2+^ concentration 5 mM, 0.1 g biochar in 20 mL solution (containing 0.01 M NaNO_3_), reaction temperature 20 °C, contact time 24 h)

Figure 5 shows the influences of initial solution pH on Ni^2+^ removal percentage and the equilibrium solution pH, as well as the fractions of Ni^2+^ removal due to precipitation calculated from the Ni (OH)_2_ solubility data using MINTEQ database. The pH_pzc_ values of all biochars, obtained from Fig. 5, are found to be within the range of 7.3–7.8, indicating that the surface of the biochars may be positively charged in slightly alkaline (7 < pH < pH_pzc_) and acidic environment, which may not aid their adsorption towards positively charged heavy metal ions due to electrostatic repulsion (Gao et al. 2013). However, on the other hand, the high pH_pzc_ and pH values suggest the strong alkalinity of the biochars which will aid their adsorption for heavy metals through surface precipitation (Inyang et al. 2015).

At pH 2, the Ni^2+^ removal percentages for all biochars are <10%. They increase as initial pH increases from 2 to 4 and stays effectively constant (around 39, 25, 20 and 13% for WSP700, WSP550, RH700 and RH550, respectively) within the pH range of 4–7. All biochars show either little change, or a slight increase (within 3%), between pH values of 7–8, and exhibit sharp increases between pH values of 8–9. All biochars reach almost completed removal (≥98%) at pH 9 and therefore exhibit no significant further change for pH 10.

The Ni^2+^ removal percentage is closely dependent on the equilibrium pH, in line with the findings of Mohan et al. (2014) and Shen et al. (2015). The equilibrium pH for all biochars increases at low initial pH values (with the range of 2–3 or 2–4) and the Ni^2+^ removal percentages correspondingly increase within this range. The increases in Ni^2+^ removal at this range are likely due to that the increase of solution pH enhances the deprotonation process of the functional groups on biochar surface and therefore create more negatively charged sites, thus increasing the adsorption of the cationic heavy metals (Qiu et al. 2008; Mohan et al. 2014). The equilibrium pH values stay almost constant through the initial pH range of 4–8; hence, the Ni^2+^ removal percentage stays nearly constant for all biochars within this range. The sharp increase in the Ni^2+^ removal between initial pH values of 8 and 9 is due to electrostatic adsorption of Ni^2+^ on biochar. At high pH values, the equilibrium solution pH exceeded the pH_pzc_ of the biochar and hence more negatively charged sites on biochar surface are created, enhancing the electrostatic adsorption Ni^2+^ on biochar (Yang and Jiang 2014). At pH 9–10, the increasing pH further enhanced the electrostatic adsorption; simultaneously, the saturation index of Ni (OH)_2_ increased (Fig. 5) significantly and therefore Ni^2+^ precipitates with OH^−^ to form Ni (OH)_2_ (Nam et al. 2015; Kadirvelu et al. 2001) which will likely be retained on the biochar surface (Inyang. 2015). It can be found from the MINTEQ analysis (Fig. 5) that the changes in solubility of Ni^2+^ did not have a significant effect on Ni^2+^ removal between pH 2 and 9.

The Ni^2+^ removal percentage at a specific pH within 2–8 follows the findings from Figs. 3 and 4, that WSP700 has the strongest adsorption capacity towards Ni^2+^ followed by WSP500, RH700 and RH500. All biochars showed the similar trend of pH dependence. At high pH values (9 and 10), the biochars exhibited completed removal of Ni^2+^ regardless of their feedstocks and production temperatures. Shen et al. (2015) observed a completed removal of Pb^2+^ at initial solution pH values between 8 and 10. Both Shen et al. (2015) and this study suggest that the adsorption of heavy metals on biochars at high pH (higher than 8 or 9) is more solution pH controlled than biochar controlled.

The buffering effect of the biochars within the pH range of approximately 4–8 suggests their potential resistance to the environmental changes in soil pH (such as groundwater flow and acid rainfall) and will therefore aid the immobilisation of heavy metals in the long term, although this resistance will be dependent on the site soil constitute, rainfall volume and biochar dosage etc. Similarly, a sharp decrease in adsorption capacity is not observed until very low pH values (approximately 3–4), which is unlikely to occur in typical soils.

### Adsorption equilibrium

**Fig. 6:**
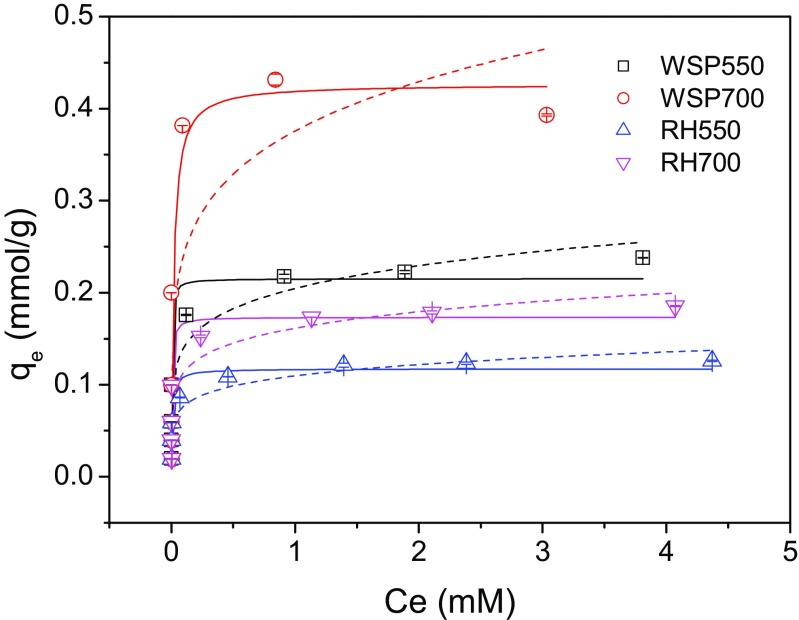
Adsorption equilibrium of Ni^2+^ fitted by Langmuir isotherm model (*straight lines*) and Freundlich model (*dash lines*) (*q*_e_—adsorption capacity at equilibrium, *C*_e_—Ni^2+^ concentration at equilibrium) (0.1 g biochar in 20 mL solution (containing 0.01 M NaNO_3_), reaction temperature 20 °C, initial solution pH 5, contact time 24 h)

The equilibrium data of Ni^2+^ are fitted to the Langmuir and Freundlich isotherm models and shown in Fig. 6 and Table 2. The *R* ^2^ values were within 0.741–0.901 for Langmuir model, which were comparable to those (0.626–0.889) for Freundlich model. According to the AIC test results, the Freundlich model was 1.465 times more likely to be correct to describe the equilibrium data compared with the Langmuir model, suggesting a heterogeneous adsorption surface for the biochars. The calculated maximum adsorption capacity of the biochars follows the order WSP700 > WSP550 > RH700 > RH550, with the value of 0.427, 0.215, 0.173, 0.117 mmol/g, which is in line with the findings in sections “Kinetics”, “Influence of solid to liquid ratio on Ni2+ adsorption” and “Influence of solution pH on Ni2+ adsorption”.

**Table 2:** Parameters and regression coefficient of the equilibrium data fitted by linear Langmuir and Freundlich isotherm models

Biochar	Langmuir			Freundlich		
	*Q*_max_ (mmol/g)	*b* (L/mmol)	*R* ^2^	*K*	1/*n*	*R* ^2^
WSP550	0.215	346.329	0.875	0.204	0.164	0.889
WSP700	0.427	52.157	0.741	0.376	0.192	0.626
RH550	0.117	121.930	0.901	0.110	0.153	0.876
RH700	0.173	247.234	0.838	0.161	0.152	0.853
AIC value	−48.778			−48.014		
Akaike weight	0.594			0.406		
AIC conclusion				1.465 times more likely to be correct		

As very limited adsorption characterisations of Ni^2+^ on biochars have been carried out to date, it is difficult to make relevant comparisons between this study and existing literature. All biochars exhibited higher maximum adsorption capacities of Ni^2+^ than Salisbury biochar (0.105 mmol/g) applied on a contaminated site in a previous study (Shen et al. 2016) as well as a more rapid adsorption of Ni^2+^ (Shen et al. 2015). In addition, these biochars exhibited higher resistances to the changing in environmental pH, which may aid the long-term stability of heavy metal immobilisation in field soil. Therefore, as Salisbury biochar was relatively successful in immobilising Ni^2+^ on this site in a 3-year preliminary study (Shen et al. 2016), these four biochars have the potential to be applied to similar sites.

### Discussion

#### Influence of biochar physicochemical properties on Ni^2+^ adsorption

The high pH dependence of Ni^2+^ adsorption on biochars suggests that the adsorption is likely controlled by electrostatic adsorption or surface precipitation, as both of them are highly pH dependent (Meena et al. 2008; Inyang et al. 2012). However, other adsorption mechanisms such as cation exchange and surface complexation cannot be excluded at this stage. Physical adsorption can be excluded as it usually lasts longer and is reversible (Inyang et al. 2015), which conflicts with the findings in kinetic and equilibrium studies.

**Fig. 7:**
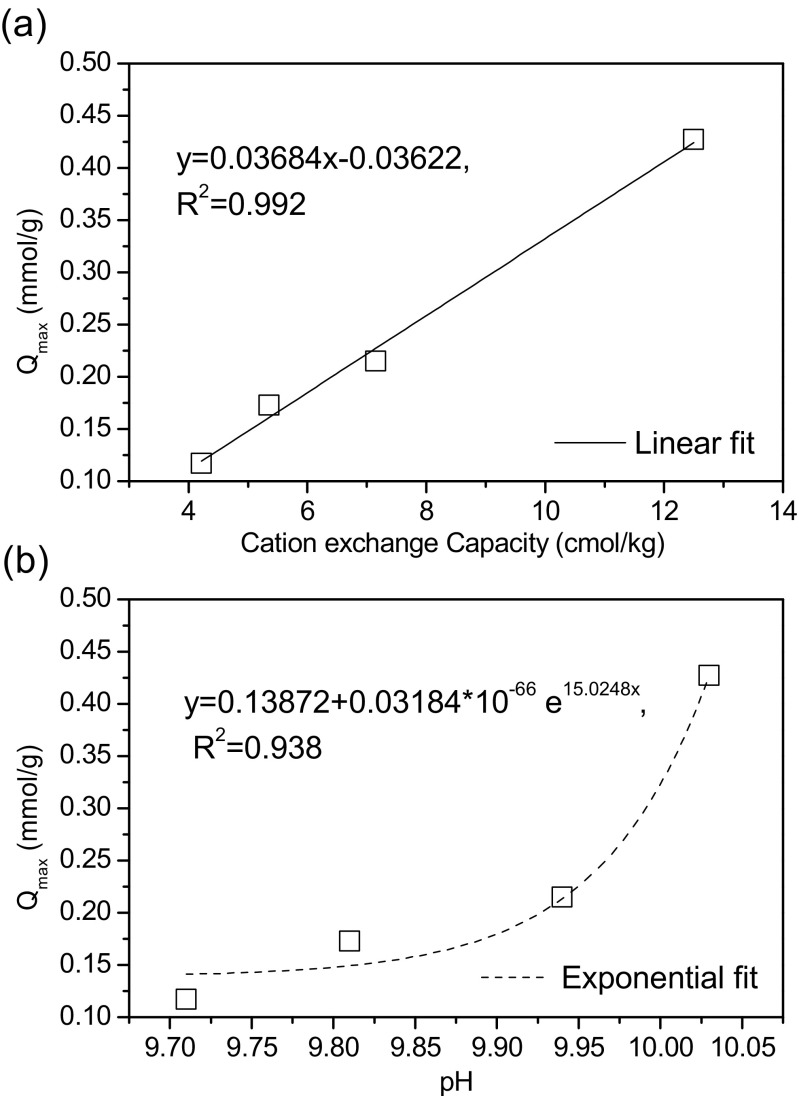
The relation between maximum adsorption capacity (*Q*_max_) of Ni^2+^ and cation exchange capacity (**a**) and pH of biochar (**b**)

In order to further explore the adsorption mechanisms, the relationship between Ni^2+^ *Q*_max_ and the physicochemical properties of the biochars are analysed and shown in Fig. S1 and Fig. 7. No significant relationships between Ni^2+^ Q_max_ and O:C value, VM content, surface area or ash content were observed (Fig. S1). The O:C value is highly related to the O-containing acidic functional groups and VM is also a strong indicator of acidic functional groups (e.g. carboxyl and phenol) (Uchimiya et al. 2011); therefore, the insignificant relationships between *Q*_max_ and VM content and O:C value suggest that the acidic functional groups and associated surface complexation (Inyang et al. 2015) may not control the Ni^2+^ adsorption on the biochars. The insignificant relationship between *Q*_max_ and surface area suggests that physical adsorption is not the predominant adsorption mechanism (Inyang et al. 2015), which is in line with the findings from kinetic study and equilibrium studies. Ash content was also not observed to have a close correlation with *Q*_max_.

In contrast, a significant positive linear relationship (*R* ^2^ = 0.992) between Ni^2+^ *Q*_max_ and CEC was observed (Fig. 6a), indicating that CEC may play an important role in Ni^2+^ adsorption. However, CEC cannot be the only mechanism controlling Ni^2+^ adsorption on the biochars. If the adsorbed Ni^2+^ through cation exchange reaches the maximum, this part will account for 33, 29, 36 and 31% of the *Q*_max_ of SWP550, SWP700, RH550 and RH700, respectively. Therefore, there must be other mechanisms controlling Ni^2+^ adsorption on the biochars together with CEC. It can be found from this study that CEC becomes a good indicator for the Ni^2+^ *Q*_max_ of biochar, and biochar with high CEC may have a high adsorption capacity of Ni^2+^.

Ni^2+^ *Q*_max_ exhibited an approximately exponential relationship (*R* ^2^ = 0.938) with the pH of biochar (Fig. 6b). The alkalinity of biochar is due to the formation of alkaline minerals (e.g. K_2_O, K_2_CO_3_) during production (Dodson 2011; Wang et al. 2014). A higher pH of biochar can result in a more alkaline environment on its surface which will favour the precipitation of Ni^2+^ on it; therefore, surface precipitation may be a mechanism controlling the adsorption of Ni^2+^ on the biochars. In addition, the formation of aromatic structure is accompanied with the formation of alkaline minerals during biochar production (Dodson 2011). A higher pH of biochar with higher alkaline mineral content also suggests a more active aromatic structure, which will aid the electrostatic adsorption (cation-π interaction) (Keiluweit et al. 2010). Therefore, this exponential relationship between Ni^2+^ *Q*_max_ and biochar pH suggests that surface precipitation and electrostatic adsorption may be two of the mechanisms controlling Ni^2+^ adsorption on the biochars.

It is of note that the alkaline minerals in biochar offers potential exchangeable cations (e.g. Na^+^ and K^+^), which has been confirmed by the CEC results (Table S1). Therefore, the CEC is highly related to the content of alkaline minerals in biochar. The good indication of CEC and pH for Ni^2+^ Q_max_ is likely both due to the alkaline minerals in the biochars.

#### The influence of feedstock type and production temperature on Ni^2+^ adsorption

As indicated across the paper, the adsorption capacity of Ni^2+^ on the biochar is highly related to its feedstock type and production temperature. WSP-derived biochar exhibited higher adsorption capacities than RH. Wheat straw and rice husk both mainly consist of cellulose, hemicellulose and lignin (Table S2). Wheat straw commonly contains more cellulose than rice husk (45.0% versus 34.5%) (Table S2), which can be easily decomposed under heating (Keiluweit et al. 2010). The decomposition process of wheat straw is shown in Dodson (2011). The carboxylate on the edge of the aromatic hydrocarbon will be decomposed and release alkaline cations (e.g. K^+^) to form alkaline minerals (e.g. K_2_O, K_2_CO_3_) and simultaneously increase the aromaticity during production. Therefore, more cellulose content may result in more alkaline minerals and higher aromaticity and consequently higher adsorption capacities for WSP derived biochar. The higher pH, CEC and K contents (Table 1) for WSP-derived biochars also support this proposed mechanism.

RH-derived biochars contain more SiO_2_ (as indicated by FT-IR spectra) and ash contents (Table 1) than WSP. Ash can block the pores on the biochar surface (Upamali et al. 2016) and therefore lower its adsorption capacity. This may also have contributed to the lower adsorption of Ni^2+^ for RH-derived biochars.

For the same feedstock, a higher production temperature will result in the formation of more alkaline minerals (Dodson 2011) (higher pH) which will aid surface precipitation and cation exchange, and more active aromatic structure which will aid electrostatic adsorption (Keiluweit et al. 2010). This explains why biochars produced at higher temperature exhibited higher Ni^2+^ adsorption capacity for the same feedstock.

In addition to biochar type, the adsorption of heavy metals on biochar depends on the properties of heavy metals such as hydrated radius, hydrolysis constant, electronegativity and relative binding strength (Sdiri et al. 2012; Sdiri et al. 2016). It is of note that this study only investigated the adsorption of Ni^2+^ on the biochars. Therefore, the adsorption of other heavy metals on the biochars may vary from Ni^2+^, which needs to be investigated in the future.

## Conclusions

In this study, the adsorption characteristics of Ni^2+^ on four standard biochars were investigated. The kinetic results show that the adsorption of Ni^2+^ on the biochars reached an equilibrium within 5 min. Greater solid to liquid ratios resulted in an increase of Ni^2+^ removal percentage but a decrease in the adsorbed amount of Ni^2+^ per weight unit of biochar. The Ni^2+^ removal percentage increased with the increasing of initial solution pH values at the range of 2–4, was relatively constantly at the pH range of 4–8, and significantly increased to ≥98% at pH 9 and stayed constantly at the pH range of 9–10. The Freundlich model was 1.465 times more likely to be correct to describe the equilibrium data compared with the Langmuir model according to the AIC test. The equilibrium study shows that WSP700 has the highest adsorption capacity for Ni^2+^, followed by WSP550, RH700 and RH550. The calculated maximum adsorption capacities of Ni^2+^ on the biochars are higher than that of Salisbury biochar which was applied to and performed well on a real contaminated site. Therefore, considering the more rapid reaction with Ni^2+^, higher resistance to changing pH and higher adsorption capacities of Ni^2+^ compared with Salisbury biochar, the standard biochars have the potential to be applied to Ni^2+^ contaminated sites.

The adsorption mechanism analysis suggests that biochar adsorbs Ni^2+^ predominantly through cation exchange, electrostatic adsorption and surface precipitation. Both pH and CEC of biochar can be a good indicator of its maximum adsorption capacity for Ni^2+^ through a positively linear and exponential relation, respectively. The differences between adsorption capacities of the biochars originate from the differences between the feedstock type and production temperature, which determine the alkaline mineral contents and associated pH and CEC, ash contents and aromatization degrees of the biochars.

This study also shows that a carefully controlled standardised production procedure makes it reliable to compare the adsorption capacities between different biochars and investigate the mechanisms involved. Analytical methods such as scanning electron microscopy with energy-dispersive X-ray analysis, X-ray diffusion analysis, X-ray photoelectron spectroscopy surface analysis, thermogravimetric analysis and sequential analysis are encouraged to be conducted for further investigation of the adsorption mechanisms. Biochars from various feedstocks are suggested to be produced according to the standard procedure of the UKBRC so that relevant comparisons can be made to identify the suitable biochars for specific contaminated sites and explore their adsorption mechanisms for heavy metals.

## Electronic supplementary material

ESM 1 (DOCX 52 kb)
