# Supplementary material for: Characteristics and mechanisms of nickel adsorption on biochars produced from wheat straw pellets and rice husk
Source: Environ Sci Pollut Res Int. 2017 Mar 31;24(14):12809–19. doi: 10.1007/s11356-017-8847-2 (PMC5418241; doi:10.1007/s11356-017-8847-2)
Supplement: Supplementary file 1 — (DOCX 52 kb) [file 11356_2017_8847_MOESM1_ESM.docx]

**Characteristics and mechanisms of nickel adsorption on biochars produced from wheat straw pellets and rice husk**

Zhengtao Shen *^, a^, Yunhui Zhang ^a^, Oliver McMillan ^a^, Fei Jin ^a^, Abir Al-Tabbaa ^a^

a (Geotechnical and Environmental Research Group, Department of Engineering, University of Cambridge, Cambridge, CB2 1PZ, United Kingdom)

* Corresponding author: Email: [ztshennju@gmail.com](mailto:ztshennju@gmail.com); [zs281@cam.ac.uk](mailto:zs281@cam.ac.uk). Phone number: +44-1223-766683. Fax: +44-1223-766683

Contents:

Table S1 Fractions of cations accounted for the CECs of the biochars.

Table S2 Chemical compositions of wheat straw and rice husk (Nepal et al. 2015).

Fig. S1. The relation between Ni^2+^ maximum adsorption capacity (Q_max_) biochar physicochemical properties: O:C (a), volatile matter content (b), surface area (c) and ash content (d).

Table S1 Fractions of cations accounted for the CECs of the biochars.

|  | WSP550 | WSP700 | RH550 | RH700 |
| --- | --- | --- | --- | --- |
| Al^3+^ | 0% | 0% | 0% | 0% |
| Ca^2+^ | 4% | 2% | 6% | 6% |
| Fe^3+^ | 0% | 0% | 0% | 0% |
| Mg^2+^ | 2% | 1% | 3% | 3% |
| Na^+^ | 29**%** | 18% | 42% | 41% |
| K^+^ | 65% | 78% | 50% | 51% |
| Mn^2+^ | 0% | 0% | 0% | 0% |

Table S2 Chemical compositions of wheat straw and rice husk (Nepal et al. 2015).

|  | Cellulose | Hemicellulose | Lignin |
| --- | --- | --- | --- |
| Wheat straw | 45.0 | 19.0 | 19.5 |
| Rice husk | 34.5 | 21.3 | 17.5 |

Fig. S1. The relation between Ni^2+^ maximum adsorption capacity (Q_max_) biochar physicochemical properties: O:C (a), volatile matter content (b), surface area (c) and ash content (d).

References

Nepal B, Chin CS, Jones S (2015) A review on Agricultural Fibre Reinforced Concrete. In: Sustainable Buildings and Structures: Proceedings of the 1st International Conference on Sustainable Buildings and Structures (Suzhou, PR China, 29 October-1 November 2015). p 125
